# HIV X4 Variants Increase Arachidonate 5-Lipoxygenase in the Pulmonary Microenvironment and are associated with Pulmonary Arterial Hypertension

**DOI:** 10.1038/s41598-020-68060-9

**Published:** 2020-07-16

**Authors:** Sharilyn Almodovar, Brandy E. Wade, Kristi M. Porter, Justin M. Smith, Robert A. Lopez-Astacio, Kaiser Bijli, Bum-Yong Kang, Sushma K. Cribbs, David M. Guidot, Deborah Molehin, Bryan K. McNair, Laura Pumarejo-Gomez, Jaritza Perez Hernandez, Ethan A. Salazar, Edgar G. Martinez, Laurence Huang, Cari F. Kessing, Edu B. Suarez-Martinez, Kevin Pruitt, Priscilla Y. Hsue, William R. Tyor, Sonia C. Flores, Roy L. Sutliff

**Affiliations:** 1 Department of Immunology and Molecular Microbiology, Texas Tech University Health Sciences Center, Lubbock, TX USA; 2 Division of Pulmonary Sciences and Critical Care Medicine, University of Colorado Anschutz Medical Campus, Aurora, CO USA; 3 Department of Medicine, Division of Pulmonary, Allergy, Critical Care and Sleep, Emory University School of Medicine, Atlanta, GA USA; 4 Atlanta Veterans Affairs Medical Center, Decatur, GA USA; 5 Department of Biology, University of Puerto Rico in Ponce, Ponce, PR USA; 6 Department of Biostatistics and Informatics, Colorado School of Public Health, University of Colorado Anschutz Medical Campus, Aurora, CO USA; 7 Department of Medicine, University of California San Francisco, San Francisco, CA USA; 8 Department of Neurology, Emory University School of Medicine, Atlanta, GA USA

**Keywords:** Molecular medicine, Pathogenesis

## Abstract

Pulmonary Arterial Hypertension (PAH) is overrepresented in People Living with Human Immunodeficiency Virus (PLWH). HIV protein gp120 plays a key role in the pathogenesis of HIV-PAH. Genetic changes in HIV *gp120* determine viral interactions with chemokine receptors; specifically, HIV-X4 viruses interact with CXCR4 while HIV-R5 interact with CCR5 co-receptors. Herein, we leveraged banked samples from patients enrolled in the NIH Lung HIV studies and used bioinformatic analyses to investigate whether signature sequences in HIV-*gp120* that predict tropism also predict PAH. Further biological assays were conducted in pulmonary endothelial cells in vitro and in HIV-transgenic rats. We found that significantly more persons living with HIV-PAH harbor HIV-X4 variants. Multiple HIV models showed that recombinant gp120-X4 as well as infectious HIV-X4 remarkably increase arachidonate 5-lipoxygenase (ALOX5) expression. ALOX5 is essential for the production of leukotrienes; we confirmed that leukotriene levels are increased in bronchoalveolar lavage fluid of HIV-infected patients. This is the first report associating HIV-*gp120* genotype to a pulmonary disease phenotype, as we uncovered X4 viruses as potential agents in the pathophysiology of HIV-PAH. Altogether, our results allude to the supplementation of antiretroviral therapy with ALOX5 antagonists to rescue patients with HIV-X4 variants from fatal PAH.

## Introduction

Today, in the days of antiretroviral therapy (ART), infection with Human Immunodeficiency Virus (HIV) is a chronic disease with almost no HIV-associated opportunistic infections and a long life expectancy^1, 2^. However, people living with HIV (PLWH) still succumb to a wide spectrum of cardiovascular complications^3–9^, including Pulmonary Arterial Hypertension (PAH)^10–13^. PAH is a life-threatening disease characterized by increased inflammatory cytokines, pulmonary vascular remodeling, vasoconstriction, and accumulation of cells that obliterate the lumina of pulmonary arteries^14, 15^. These pathological events contribute to significant increases in pulmonary artery pressures, eventually leading to right ventricular hypertrophy and right heart failure.

It is well recognized that PAH is significantly more frequent in PLWH than in uninfected people^10–13, 16, 17^. Although the mortality rate is high, how HIV causes or contributes to the pathophysiology of HIV-PAH remains a mystery. HIV replicates effectively in the lungs^18, 19^ but there is no definitive proof that HIV directly causes PAH. However, it is well established that HIV proteins such as Tat, Nef and glycoprotein 120 (gp120) play significant roles in pathogenic pulmonary vascular remodeling and HIV-PAH^20–27^. The interactions of viral proteins with molecular partners in the infected cells induce inflammation and deregulate apoptosis and proliferation of vascular endothelial cells in the lung, resulting in pulmonary vascular remodeling^24, 25, 28–34^. Our group showed that HIV Nef protein co-localizes with pulmonary endothelial cells in PAH-like plexiform lesions and is associated with cardiac hypertrophy and inflammatory markers consistent with PAH in a macaque model^35–37^. Moreover, we found specific HIV Nef polymorphisms in patients with HIV-PAH^20^.

The mere presence of HIV in the lungs may still impact the pulmonary cell biology via the co-receptors used to infect the cells. HIV enters susceptible cells via interactions of the viral gp120 with the host CD4 receptor and either the C–C chemokine receptor-5 (CCR5) or the C-X-C chemokine receptor-4 (CXCR4)^38–40^. The HIV isolates that use the CCR5 as co-receptors are widely known as R5, while those that use the CXCR4 co-receptors are known as X4. Binding of HIV or natural ligands to either CCR5 or CXCR4 G-protein coupled receptors activates signaling pathways producing inflammation and cytotoxicity^41, 42^. Endothelial cells are mostly refractory to HIV infection however, the presence of HIV gp120 activates and triggers apoptosis and dysfunction in these cells^28, 31, 43, 44^. This study sought to gain insights into the potential role of HIV gp120 in PAH. Because gp120 determines HIV tropism^40^ and can trigger different signaling pathways upon interactions with specific co-receptors, we leveraged multiple HIV models including existing peripheral blood samples from patients with HIV-PAH enrolled in the NHLBI Lung HIV studies, in vitro approaches, HIV transgenic rats, and bronchoalveolar lavage fluids from PLWH to test the hypothesis that signature sequences in the HIV *gp120* gene will reveal specific viral tropism in patients with HIV-PAH^45–47^.

## Results

### HIV gp120^X4^ variants are significantly over-represented in people with HIV-PAH

We analyzed peripheral blood samples from 39 PLWH enrolled in the NIH/NHLBI Lung HIV Studies at the University of California—San Francisco (UCSF) and University of Colorado (CU-Anschutz), who underwent evaluation for PAH. The subjects enrolled in this study featured a mean age of 52 years old, mostly males (n = 32, 82%), with mean length of infection of 16 years from the HIV diagnosis until consented sample collection. Most of the individuals were treated with antiretroviral therapy (n = 32, 82%); 43% had HIV viral loads below the limits of clinical detection (< 40 copies/mL), with CD4^+^ cell counts ranging from 77 to 1,410 cells/µl and mean CD4 counts = 575 cells/µl (Table 1). Using 200 CD4 cells/µl as cutoff for AIDS diagnosis, we had 6 AIDS patients in this study. Diagnoses of HIV-associated PAH were made based on the standard diagnostic algorithm, which includes Doppler echocardiography and right heart catheterization (RHC) if echocardiography suggested PAH. Based on hemodynamic (mPAP by RHC) and echocardiographic data (PASP), this study includes 16 pulmonary hypertensive and 23 normotensive subjects (Table 2).

**Table 1:** General description of the HIV-infected patient cohort.

Parameter	Number of subjects analyzed	Mean	SD	Median	Min	Max
Age (years)	39	52	7	52	38	62
Duration of HIV infection (years)	39	16	7	17	2	30
HIV viral load (copies/µL)	39	7,863	24,497	72	40	112,387
CD4 counts (cells/µL)	35	574	271	511	77	1,410
PASP (mm Hg)	39	41	18	35	18	94
mPAP (mm Hg)	31	27	13	25	15	60

A total of 39 HIV-infected informed consenting subjects were analyzed. PASP were measured by echocardiography in all subjects. The mPAP were determined by right heart catheterization in 31 of the subjects. Data were summarized using descriptive statistics using GraphPad Prism 6.

**Table 2:** Summary of demographic and clinical characteristics of HIV-infected participants stratified by pulmonary disease status.

Characteristic	Statistic	Normotensive	Pulmonary hypertensive	Total
**Gender**				
Male	n (%)	19 (59%)	13 (41%)	32 (82%)
Female	n (%)	4 (57%)	3 (43%)	7 (17%)
**Ethnicity**				
African-American	n (%)	8 (38%)	6 (37%)	14 (37%)
Caucasian	n (%)	9 (42%)	10 (62%)	19 (51%)
Other	n (%)	4 (19%)	–	4 (10%)
**CD4 counts**				
Asymptomatic	n (%)	21 2(91%)	12 (75%)	33 (84%)
AIDS	n (%)	2 (8%)	4 (25%)	6 (15%)
**Antiretroviral therapy**				
Naïve	n (%)	5 (21%)	2 (12%)	7 (17%)
Experienced	n (%)	18 (78%)	14 (87%)	32 (82%)
**HIV viral loads**				
Viremic	n (%)	13 (56%)	9 (56%)	22 (56%)
Suppressed	n (%)	10 (43%)	7 (43%)	17 (43%)
**Hemodynamics**				
mean PAP (by RHC)	Mean ± SD	18.20 ± 2.8	36.06 ± 11.9	
Mean PASP (by echo)	Mean ± SD	33.57 ± 6.4	51.19 ± 23.5	

We grouped the research subjects enrolled into this study as *per* the following criteria: pulmonary normotensive (mPAP < 25 mm Hg by RHC or PASP by echocardiography if RHC was not performed), pulmonary hypertensive (mPAP > 25 mm Hg by RHC or PASP by echocardiography if RHC was not performed); asymptomatic for HIV disease (CD4 counts > 200 cells/ul); AIDS (CD4 counts < 200 cells/ul); antiretroviral treatment naïve if they reported having no experience with antiretroviral therapy any time on or before enrollment into this study; viremic if patient had HIV viral loads > 40 copies/ml at sampling time or 6 months before and; suppressed if patient had HIV viral loads < 40 copies/ml at sampling time or 6 months before.

**Figure 1:**
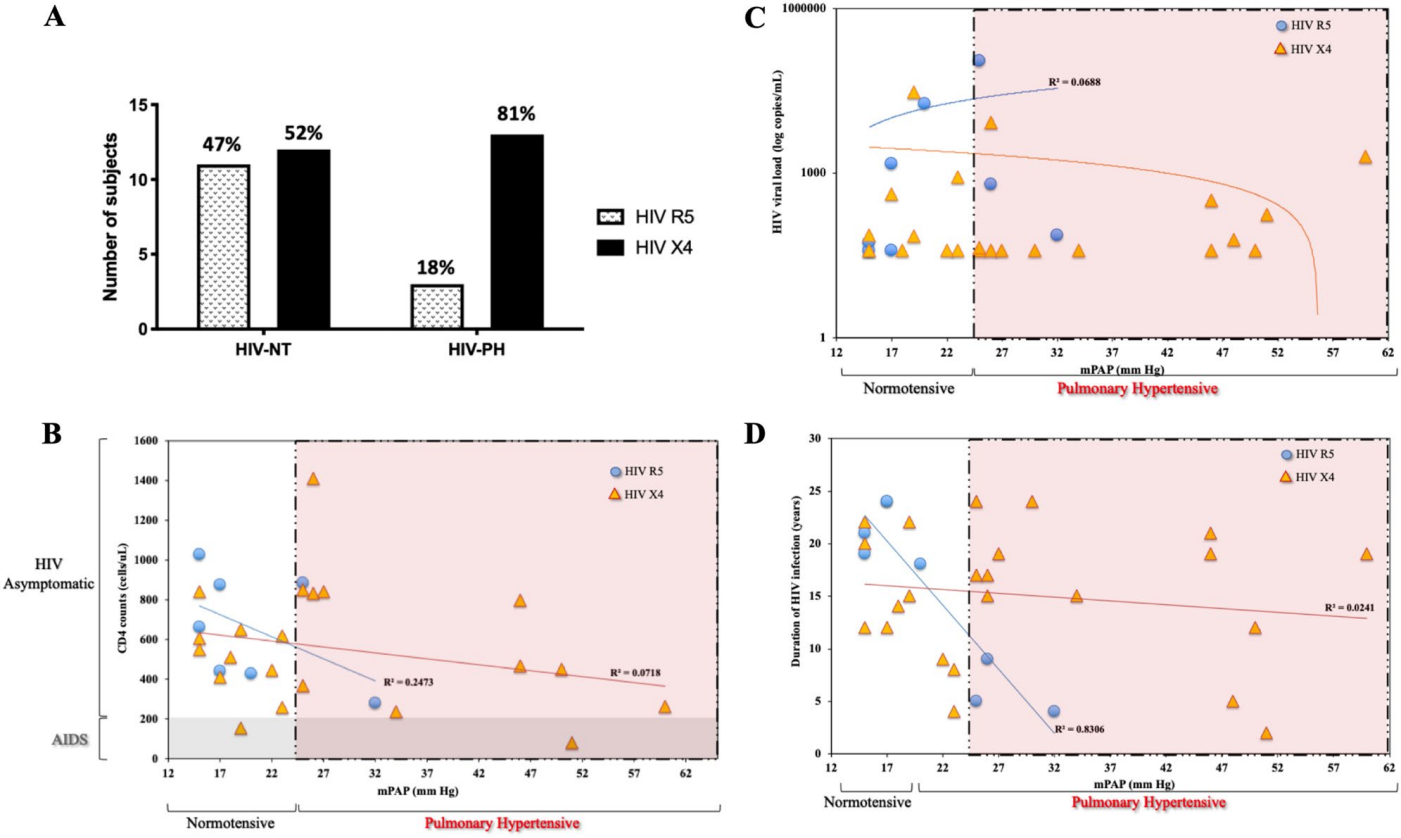
HIV X4 viruses are over-represented in asymptomatic HIV patients with pulmonary hypertension. (**a**) We used translated sequences of the V3 region of the HIV-1 *envelope* gene to predict HIV tropism (co-receptor usage) by using the bioinformatics tool Geno2Pheno^46^. Asymptomatic patients were defined as CD4 counts > 200 cells/µl. Pulmonary hypertensive patients were defined as those with mean mPAP > 25 mm Hg by RHC or PASP > 25 mm Hg by echochardiography, while normotensive patients were defined as patients with mPAP < 24 mm Hg or PASP < 24 mm Hg. The percentages (%) on top of the bars indicate the percentage of patients harboring either HIV R5 or X4 variants. (**b–d**) Correlations between mPAP and CD4 counts (**b**), HIV viral loads (**c**), and duration of HIV infection (**d**)**.** Each symbol represents a subject. Patients harboring HIV-R5 are indicated in blue circles; patients with HIV-X4 are indicated with yellow triangles. All CD4 counts < 200 cells/ul in the AIDS zone are shaded in gray (Panel B), while the mPAP within the PH zone are shaded in pink.

Our previous studies associated the presence of specific Nef polymorphisms with HIV-PAH^20^. The promiscuous nature of Nef interactions with cellular protein partners triggers cell signaling pathways leading to vascular dysfunction. In addition, the binding of HIV to receptors on the cell surface induces cellular dysfunction, via its envelope gp120 protein. Therefore, we sought to characterize the HIV-*gp120* gene in the context of PAH. We sequenced the tropism-defining C2-V4 region of the HIV- *gp120* gene from the enrolled patients and generated a total of 1,034 HIV-*gp120* molecular clones. Reconstruction of phylogenetic trees for HIV-*gp120* showed that all the clones clustered by patient, suggesting that there was no cross-contamination between the molecular clones (data not shown). For consistency purposes, only patients infected with HIV subtype B strains were included in our analyses for consistency purposes. To investigate whether PAH is associated with signature sequences in HIV *gp120*, we analyzed the presence of specific aminoacid sequences which predict HIV co-receptor usage in the V3 loop of HIV gp120, which serve as benchmarks to predict HIV co-receptor, by using the genotypic analysis tool Geno2Pheno^47^. The data showed that 13 out of 16 subjects with HIV-PAH (81%) harbored HIV-X4 strains. Pulmonary normotensive (NT) individuals (n = 23) showed almost an equal distribution of subjects harboring X4 or R5 strains: 12 individuals (52%) had HIV-X4 and 11 HIV-NT subjects (47%) had HIV-R5 (Fig. 1a).

The appearance of HIV-X4 viruses is generally associated with advanced HIV infection, delayed viral suppression, and AIDS^38, 48–50^; nevertheless, X4 viruses have also been associated with development of non-AIDS events like hypertension, renal dysfuction and metabolic and bone disorders ^51^. In this study, we stratified the dataset by disease stage (asymptomatic HIV or AIDS). The results showed that HIV asymptomatic individuals with CD4 > 200 cells/µL and diagnosed with HIV-PAH (defined mPAP ≥ 25 mm Hg by RHC) harbored X4 viruses more frequently than HIV-NT (*p* = 0.0039). Our cohort included six AIDS patients; however, we did not observe an over-representation of X4 viruses in our AIDS patients (CD4 counts < 200 cells/µL, *p* = 0.92, Fig. 1b). Based on statistical analyses, the odds of PAH were 5.5 times higher in HIV-asymptomatic (non-AIDS) subjects with X4 viruses than the odds of PAH in patients with R5 (OR = 5.50, 95% CI 1.75–17.24). Studies from Santoro, et al.^52^ suggest that HIV-*gp120* V3 genotypic analyses using 2% as cutoff for false positive rate (FPR), as opposed to FPR 10% better define viral populations capable of greater cytopathic effects, and more advanced disease. To increase the level of stringency of our analyses, we also used FPR ≤ 2% as cutoff. In this case scenario, 27 out of 39 of the study subjects (69%) had FPR values ≤ 2% (thus, were predicted to harbor HIV-X4 strains), while 12 out of 39 (31%) were predicted to harbor HIV-R5 viruses. In the context of pulmonary phenotype, we had hemodynamic data from 31 out of the 39 subjects. We found that 6 individuals with HIV-PAH (38% of the cohort) were predicted to harbor HIV-R5 viruses and 10 (62%) harbored HIV-X4, while 14 HIV-NT individuals (93%) had HIV-R5 and one had HIV-X4. Similar to the analyses using FPR 10% as cutoff, we confirmed that asymptomatic non-AIDS patients with HIV-PAH harbored significantly more HIV-X4 (*p* = 0.0121) but not AIDS patients (*p* = 0.41). These data indicate that PLWH and with a predominance of X4 viruses are more likely to exhibit a PAH phenotype without progressing to AIDS. Further analyses showed no statistically significant differences in the viral loads nor in the duration of HIV infection in patients harboring either X4 or R5 viruses (*p* = 0.64, Fig. 1c, d).

### HIV glycoprotein variants R5 and X4 induce distinct effects on apoptosis and proliferation in human pulmonary endothelial cells in vitro

**Figure 2:**
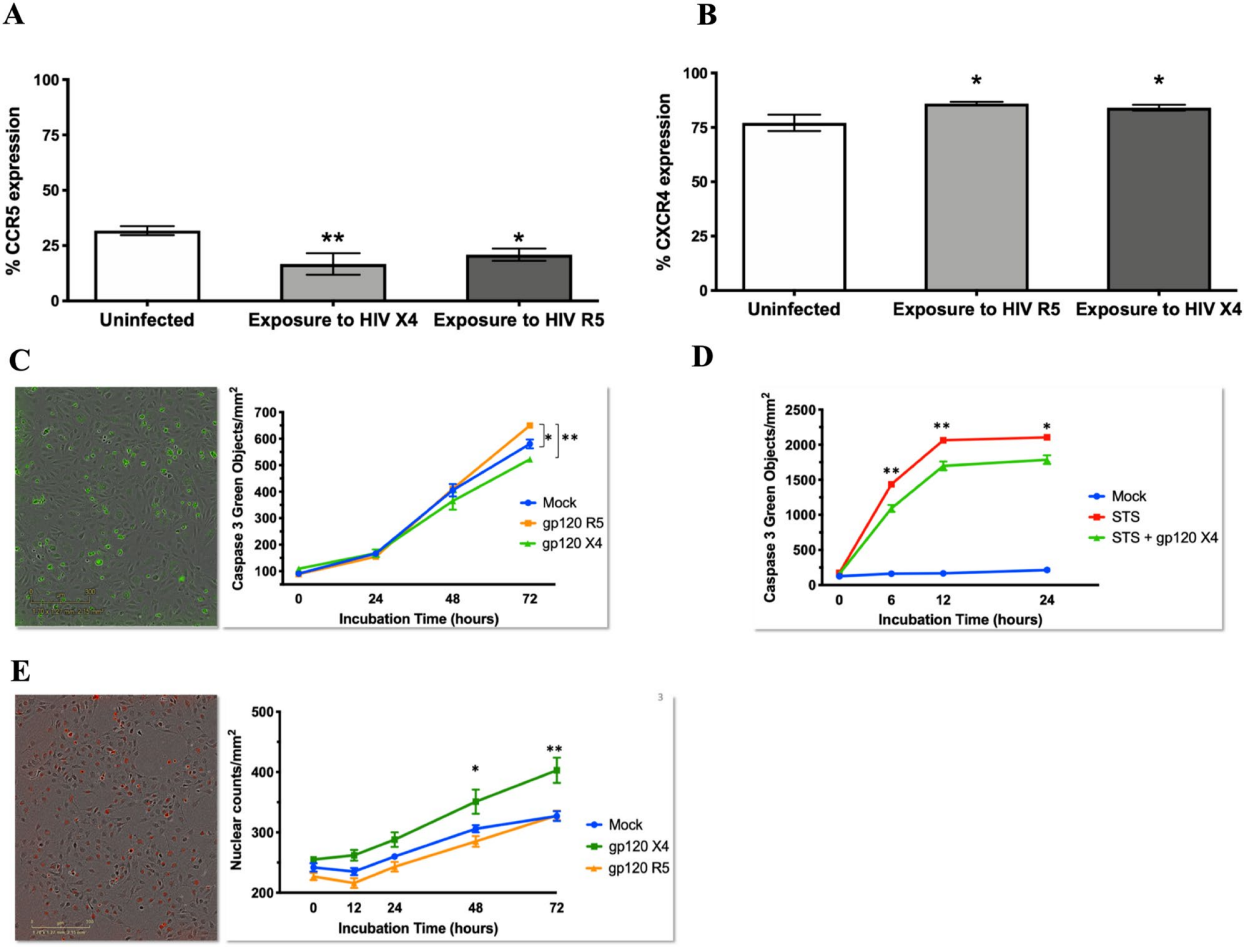
HIV glycoprotein variants have different apoptotic effects in cultured pulmonary endothelial cells. Pulmonary endothelial cells express the HIV chemokine co-receptors CCR5 and CXCR4. HPAEC were cultured in the presence of uninfected or HIV-infected T lymphocytes for 48 h. Cells were stained with anti-human CXCR4 (**a**) and anti-human CCR5 (**b**) antibodies and analyzed by flow cytometry. (**c**) HIV gp120 R5 induce pro-apoptotic effects in HPAEC. HPAEC were treated with 500 ng/ml of recombinant HIV glycoproteins (NIH AIDS Reagents Program) or vehicle for 24 or 48 h (n = 8 for each condition), in the presence of a caspase-3/7 non-fluorescent, cell permeant (DEVD) substrate (CellPlayer, Essen Biosciences). Activated caspase 3/7 cleaves the DEVD substrate to release a DNA-intercalating green fluorescent signal. Green-labeled nuclei were reported as green nuclei counts per mm^2^ using an IncuCyte ZOOM Imager (Essen Biosciences). Stars indicate statistically significant differences between the groups indicated by brackets. (**d**) Anti-apoptotic effects of HIV gp120 X4 on cultured HPAEC. HPAEC were treated with 0.1 uM staurosporine (STS) alone or combined with 500 ng/ml recombinant HIV R5 or X4 glycoproteins for 12 or 24 h, in the presence of caspase 3/7 substrate as described (n = 4 for each condition). The appearance of apoptotic bodies was quantified as green nuclei counts per mm^2^ in real time using the IncuCyte ZOOM. Stars indicate statistical significance of STS group compared to STS + gp120 treatment. (**e**) HIV gp120 X4 induce proliferative phenotypes in pulmonary endothelial cells. HPAEC were treated with 500 ng/ml recombinant HIV glycoproteins or vehicle for 24, 48, or 72 h (n = 8 for each condition) in the presence of a red fluorescent nuclear intercalating agent (NucLight Red, Essen Biosciences). Nuclear counts were quantified as red fluorescent objects per mm^2^ in real time using the IncuCyte ZOOM. In all datasets, stars indicate statistical significance: *p < 0.01; **p < 0.001; all panels show the data as mean, SEM.

Vascular endothelial cells are resistant to HIV infection^44, 53, 54^ but surrender to the cytopathic effects of HIV as bystander cells. Our flow cytometry analyses of the HIV co-receptor expression on human pulmonary artery endothelial cells (HPAEC) showed that 12–33% of these cells express CCR5 (Fig. 2a) and that 73–87% of the cells express the CXCR4 (Fig. 2b) chemokine receptors that by flow cytometry. Altogether, these results confirm that pulmonary endothelial cells express both HIV co-receptors CCR5 and CXCR4, which may contribute to the endothelial susceptibility to HIV effects.

There is evidence in the literature that HIV glycoproteins induce apoptosis on HPAEC^28^, with some studies comparing the direct effects of R5 vs X4^28, 55–57^. Here, we investigated whether R5 and X4 HIV gp120 induce differential biological effects on pulmonary endothelial cells in vitro. We exposed the cells to physiological concentrations of HIV recombinant gp120 R5^BaL^ or gp120 X4^IIIb^ at 500 ng/mL^58^, using active caspase 3/7 as readout of apoptosis (Fig. 2c). Compared to control cells, we observed significant increases in caspase 3/7 activity in HPAEC treated with gp120^R5^ after 72 h (*p* < 0.01). We also found significantly decreased caspase 3/7 activity in gp120^X4^ protein-treated cells compared to mock after 72 h (*p* < 0.001). The finding that gp120^R5^ but not gp120^X4^induced significantly more apoptosis on HPAEC suggest the two variants of HIV glycoproteins have a differential effect on apoptotic activity in cultured HPAEC.

Intrigued by the significantly lower number of apoptotic bodies in HPAEC treated with X4 glycoprotein, we examined whether HIV glycoprotein induces apoptosis-resistant phenotypes in HPAEC. We treated HPAEC with the protein kinase inhibitor staurosporine (STS) in the presence of either HIV glycoprotein R5 or X4. Figure 2d shows that HPAEC treated with STS exhibited significantly increased caspase 3/7 activity after 6 h than cells treated with STS mixed with HIV glycoproteins (*p* < 0.001). In addition, cell proliferation assays showed that cells treated with gp120^R5^ did not differ from mock-treated cells after 72 h treatments. However, cells exposed to HIV gp120^X4^ displayed a statistically significant increase in proliferation at 48 h (*p* < 0.01) and 72 h (*p* < 0.001, (Fig. 2e).

Expression of recombinant HIV gp120 stimulates pulmonary endothelial 5-lipoxygenase expression in vitro.

**Figure 3:**
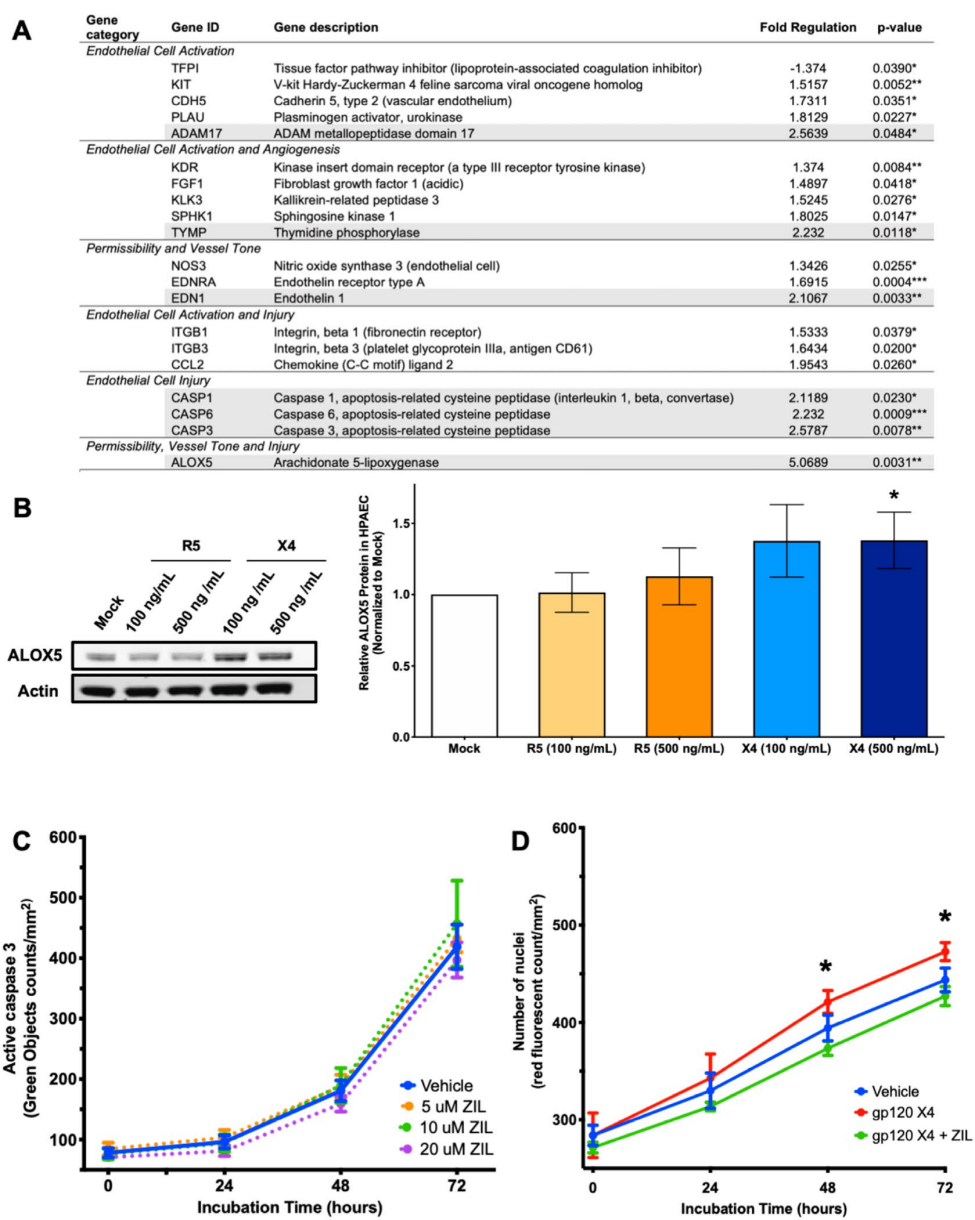
HIV gp120 stimulate expression of genes associated with endothelial cell activation and constrictive mediators. (**a**) HIV gp120 induces genetic changes in pulmonary endothelial cells. HPAEC were cultured with 500 ng/mL of recombinant gp120-X4_IIIb_ for 16 h. Endothelial gene expression was analyzed by using the Endothelial Cell Biology PCR array (Qiagen). Only statistically significant fold changes are shown. Genes with > twofold regulation are highlighted in gray. Note the fivefold increased expression of vasoconstrictive arachidonate 5-lipoxygenase (ALOX5). (**b**) Confirmation of gp120 X4-induced increased expression of pulmonary ALOX5 by Western blot. HPAEC were treated with media containing 500 ng/mL of either R5 or X4 gp120. The cells were grown for 72 h with media replacement every 24 h. The increased expression of ALOX5 was confirmed by immunoblotting, using actin as internal control. Densitometry analyses are shown after normalization with actin and mock samples. (**c**) ALOX5 inhibitor Zileuton is not toxic to endothelial cells. We tested different concentrations of the ALOX5 inhibitor Zileuton in cultured HPAEC, using apoptosis as a readout for cellular toxicity. Adherent HPAEC were treated with media containing 5–20 µM of Zileuton, incubated for 48 h and analyzed for expression of active caspase 3. No statistically significant differences were observed in activation of apoptosis among all treatments, compared to vehicle control. (**d**) Zileuton decreases HIV gp120-induced proliferation in HPAEC. We analyzed HPAEC proliferation in the presence of HIV gp120 with and without Zileuton for 48 h, using nuclear counts as readout. For all datasets: *p < 0.05 when compared to vehicle/mock control.

We examined whether HIV *gp120* variants have a differential impact on pulmonary artery endothelial gene expression. To this end, we cultured HPAEC in the presence of recombinant HIV gp120^X4^ protein to analyze gene expression using the Endothelial Cell Biology PCR array (Qiagen). We found that HIV gp120^X4^ significantly dysregulated the expression of 20 of the 84 genes in the panel; the genes were mainly associated with angiogenesis and EC injury (Fig. 3a). Of these 20 genes, 7 genes had a > twofold regulation, most notably arachidonate 5-lipoxygenase (ALOX5, fivefold increased, *p* < 0.01). ALOX5 is essential for production of biologically active leukotrienes. The increased protein expression of *ALOX5* was confirmed by Western blot (Fig. 3b).

Our results align well with documented associations between increased ALOX5 protein levels and PAH^59^. Treatment with MK886 (inhibits FLAP, 5-Lipoxygenase Activating Protein) and zileuton (inhibits ALOX5) prevents PAH in experimental models^60, 61^. Overexpression of ALOX5 markedly enhances the increase in RVSP in response to monocrotaline (MCT) treatment in rats, and ALOX5 inhibition attenuated elevations in RVSP^60^. These results suggest that endogenous ALOX5 mediates MCT-induced PAH and increased ALOX5 expression may exacerbate PAH pathologies. ALOX5 is present in HPAEC. ALOX5 and FLAP inhibitors, with EC_50_ ranging between 0.5 and 5 µM, are able to dose-dependently prevent cell growth without inducing cell cytotoxicity^62^. Taken together, evidence indicates that ALOX5 play a critical role in PAH pathogenesis and our observations point to a potentially novel role of HIV gp120^X4^ in PAH, via ALOX5.

### ALOX5 inhibitor Zileuton reverses HIV gp120^X4^-induced endothelial proliferation

Zileuton is a 5-lipoxygenase inhibitor that is routinely used for clinical prophylaxis and long-term maintenance of obstructive airway diseases like asthma^63^. To investigate whether increased ALOX5 expression mediated by HIV gp120^X4^ contributes to endothelial cell dysfunction, we exposed cultured HPAEC to recombinant HIV gp120^X4^ in the presence or absence of Zileuton. First, we tested whether Zileuton is safe to use on endothelial cells by treating the cells with media containing 5, 10, or 20 uM of Zileuton for 72 h, while collecting data on the expression of active caspase 3 via live cell imaging. We found that HPAEC did not undergo apoptosis with any of the doses tested (Fig. 3c), suggesting it is safe for endothelial cells. Because HIV gp120^X4^ induced cell proliferation and increased ALOX5 expression in HPAEC, we further investigated whether these effects were related. To this end, we examined HPAEC proliferation in the presence of HIV gp120, with and without Zileuton for 72 h. Our results confirmed gp120^X4^ mediated increases in HPAEC proliferation and moreover, that treatments with 10 uM of Zileuton reversed the increased HPAEC proliferation induced by HIV gp120 ^X4^ (Fig. 3d).

### HIV expression increases pulmonary endothelial 5-lipoxygenase expression in vitro

**Figure 4:**
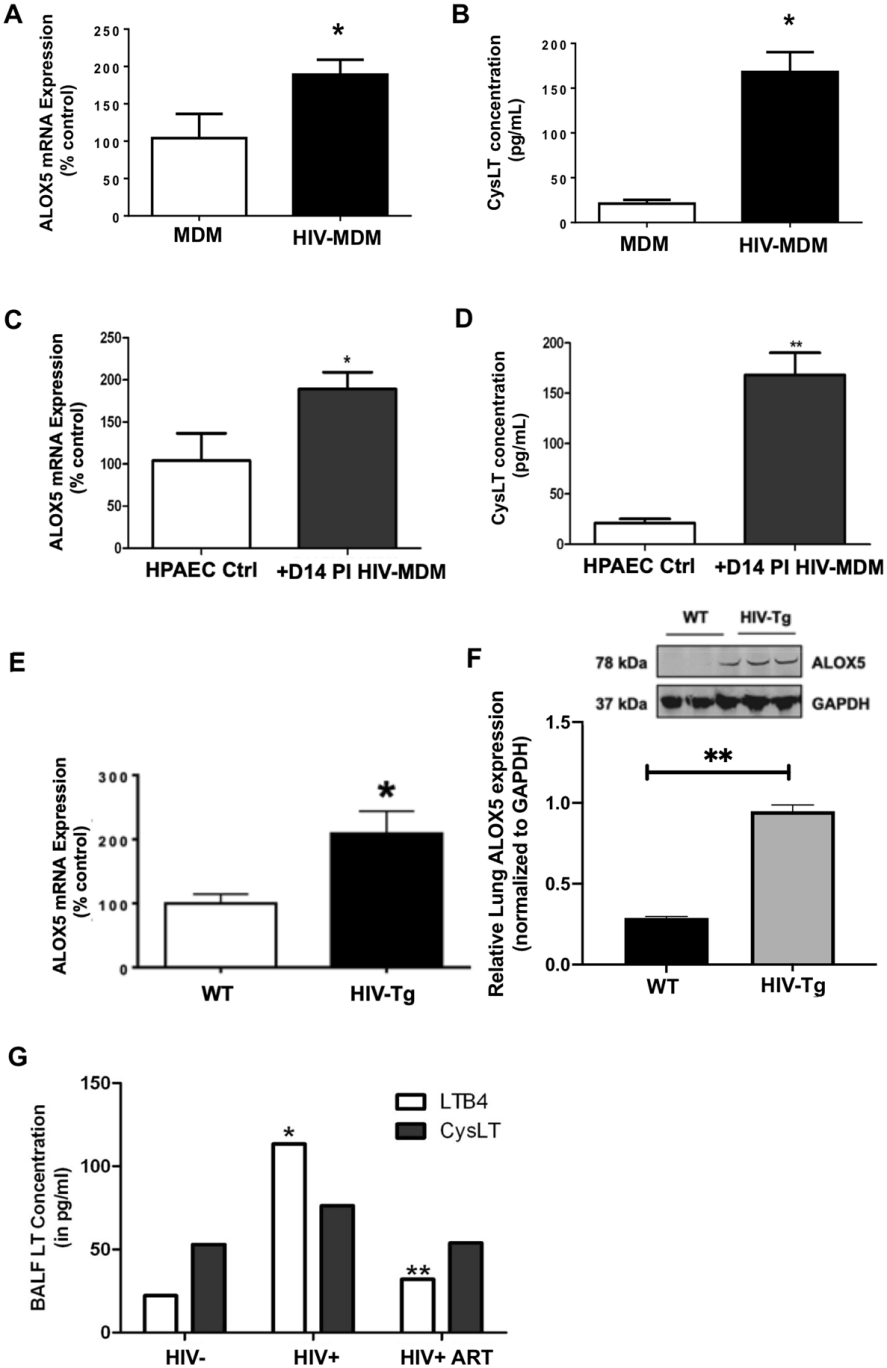
Infectious and transgenic HIV expression increase the expression of ALOX5 and release of leukotrienes (**a**) HIV increases the expression of ALOX5 and (**b**) Cysteinyl leukotrienes (Cys-LT) in monocyte-derived macrophages (MDM). MDM were cultured with HIV-1_ADA_ for 1 h and processed for detection of ALOX5 mRNA expression by quantitative real time PCR and CysLT by ELISA (n = 5 for each condition). (**c**) Increased expression of ALOX5 and (**d**) CysLT in cultured HPAEC cultured with HIV-MDM conditioned media for 24 h. HIV-MDM medium was diluted to clinically-relevant levels of p24 or 50 pg/ml^98^. (**e**) HIV-1 transgene increases ALOX5 expression in vivo. HIV transgenic rat lungs were evaluated for the expression of ALOX5 mRNA by quantitative real time PCR, compared to wild-type rats, using GAPDH as internal control. The relative expression of ALOX5 in rat lungs is expressed as percent of control. (**f**) The increased expression of ALOX5 was confirmed by Western blot, normalized to GAPDH. For all datasets: *p < 0.05 when compared to control. **p < 0.001 when compared to controls. (**g**) HIV antiretroviral therapy reverses HIV-induced increase in leukotriene levels in bronchoalveolar lavage fluid. We measured the levels of leukotriene LTB_4_ (n = 3–4) and cysteinyl leukotrienes (CysLT, n = 6–10) in bronchoalveolar lavage fluid (BALF) from uninfected (HIV-), HIV-1 positive (HIV+), and HIV+ patients receiving antiretroviral therapy (HIV + ART). *p < 0.01 when compared to BALF from control, uninfected subjects. **p < 0.001 when compared to HIV+ subjects.

Endothelial cells are largely believed to be resilient to HIV infection yet multiple studies demonstrate that HIV significantly impairs endothelial cell function as bystander, uninfected entities^64, 65^. To determine whether infectious HIV induces these effects by altering endothelial gene expression, we sought to culture HPAEC in conditioned medium from HIV-infected and uninfected monocyte-derived macrophages (MDM) for 24 h. Our results show that HIV infection of MDM alone significantly increased the expression of ALOX5 and release of cysteinyl leukotrienes (Fig. 4a, b, *p* < 0.01). Cultures of HPAEC in HIV + MDM conditioned media also showed significant increases in the expression of ALOX5 (*p* < 0.01) and release of cysteinyl leukotrienes (*p* < 0.001) in HPAEC (Fig. 4c, d).

### HIV expression increases pulmonary endothelial 5-lipoxygenase expression in vivo

HIV transgenic (Tg) rats are known to exhibit significant increases in ALOX5 mRNA and protein expression in the brain, compared to wild-type controls^66^. It is also well established that HIV Tg rats develop pulmonary arterial hypertension with increased vascular remodeling^32, 67–71^. Here we sought to determine whether HIV transgene expression alters pulmonary ALOX5 expression in vivo. We found that HIV Tg animals exhibited a twofold increase in pulmonary ALOX5 expression when measured using quantitative real-time PCR (*p* < 0.01) and Western blot analyses, *p* < 0.01 (Fig. 4e, f), suggesting that HIV protein expression increases pulmonary ALOX5 expression in vivo.

### Leukotriene levels are increased in bronchoalveolar lavage fluid of PLWH

The enzyme 5-lipoxygenase, which is coded by ALOX5, catalyzes the conversion of arachidonic acid to biologically active leukotrienes, including leukotriene B4 (LTB4) and cysteinyl leukotrienes (CysLT). To determine whether HIV infection alters leukotriene levels in the lung, we measured LTB_4_ and CysLT in BALF from uninfected (control) subjects, HIV-infected patients (HIV +), and HIV-infected patients on antiretroviral treatment (HIV + ART). We found that untreated HIV + patients had significantly higher levels of LTB4 ( *p* < 0.01) and this increase was not observed in patients receiving ART (Fig. 4g). These results suggest that HIV replication drives the increased leukotrienes release in the lungs.

### HIV gp120 ^X4^ alters the ERK signaling pathway in lung bronchial cells

**Figure 5:**
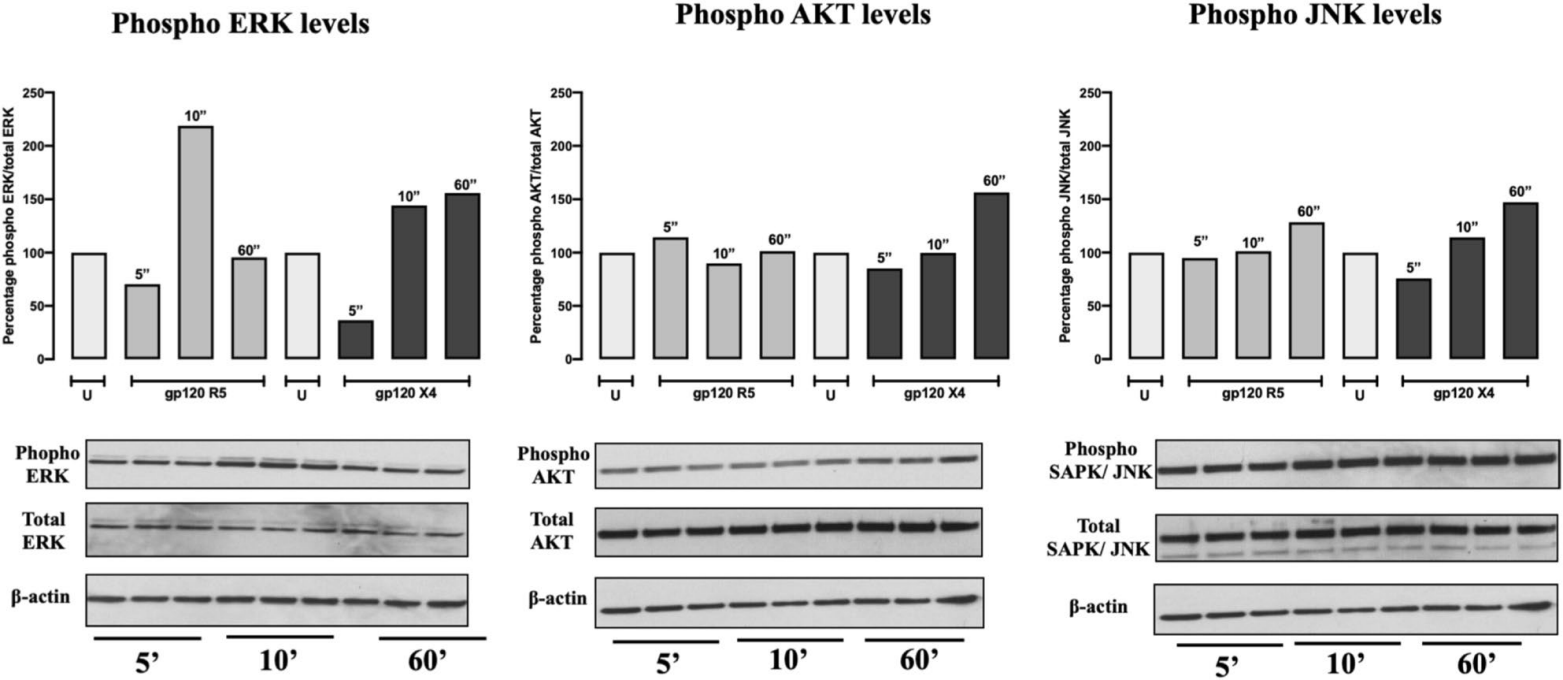
Bronchial epithelial cells exhibit extended phosphorylation states after treatment with HIV gp120 X4. Cultured HBE4 cells were exposed to 500 ng/ml of HIV proteins or vehicle for 5–60 min at 37 °C and subsequently immunoblotted for total and phosphorylated (**a**) ERK, (**b**) AKT, and (**c**) SAPK-JNK by Western blot, with beta actin used as internal control. Results are presented as percentage of phosphorylated protein over total protein. Incubation times (minutes) are presented on top of each bar.

Studies by Brune and co-workers established that HIV X4 can initiate and maintain local inflammation after significant activation of extracellular signal-regulated kinase (ERK)^72^, leading to increased lung epithelial permeability and potential implications in chronic lung disease. Guided by their and our findings, we further investigated whether our findings in the pulmonary vascular compartment potentially mirror X4-induced changes in the pulmonary epithelial compartment and how it compares to R5. We cultured normal lung epithelial cells (HBE4) in the presence of recombinant HIV gp120 X4 or R5 and measured the expression levels of phosphorylated ERK, Protein Kinase B (AKT) and C-Jun N-terminal Kinase (JNK) by Western blot. We found that HIV gp120 X4 (but not R5) exhibited a consistent increase in all ERK, JNK and AKT phosphorylated molecules overtime and that both glycoproteins increased JNK phosphorylation (Fig. 5). Our results support that gp120 X4 activates phosphorylation cascades in classic mitogenic signaling pathways, potentially leading to pathological changes in the lung epithelial cells.

## Discussion

Pulmonary arterial hypertension associated with HIV infection remains an enigmatic disease. Several lines of evidence highlight the role of HIV proteins in the pathophysiology of PAH. Nonetheless, deeper mechanistic insights are still needed to better understand the host-virus interactions affecting the pulmonary vasculature. Our previous work on HIV Nef documented polymorphisms that may change the protein landscape and affect interactions of Nef with host molecular partners^20^. Herein, we determined the role of HIV *gp120* variants in PAH by leveraging banked blood samples of PLWH with and without PAH; we learned that significantly more persons living with HIV-PAH harbor HIV-X4 variants, while the distribution of R5 and X4 viruses was almost equal in normotensive individuals.

PAH is a complex disease of the pulmonary vasculature. Vascular endothelial cells are known to be resistant to HIV infection, as HIV has not been isolated from endothelial cells of patients who develop PAH^73, 74^ nor has HIV DNA, RNA, or p24 antigen been detected in the pulmonary endothelium after exposure to HIV^44, 53, 54^. Here we confirmed that pulmonary endothelial cells express the HIV chemokine co-receptors CCR5 and CXCR4, which aligns well with the literature^75–77^ and supports the principle that these cells succumb to the cytopathic effects of HIV as pulmonary bystander entities in the absence of infection. Some studies have compared the effects of HIV glycoproteins R5 and X4^28, 55, 78^. Our comparative studies in vitro showed that HIV-R5 glycoprotein has apoptotic effects on pulmonary vascular cells as opposed to the gp120^X4^ protein, which promoted apoptosis-resistant and proliferative phenotypes in the cells. In this study, we also observed that recombinant gp120^X4^ as well as infectious HIV-X4 virus elicit remarkable increase in gene and protein expression of ALOX5 in multiple HIV models. In addition, our results in HIV transgenic rats showed that HIV protein expression increased pulmonary ALOX5. Because ALOX5 is an essential mediator in the production of biologically active leukotrienes, we investigated bronchoalveolar specimens of HIV Tg rats and PLWH and confirmed that indeed, leukotrienes levels are increased in BALf in vivo. Altogether, these results suggest that HIV expression increases pulmonary genetic expression of ALOX5, which leads to over-activation of the pro-inflammatory 5-lipoxygenase system with subsequent increases in leukotriene formation.

**Figure 6:**
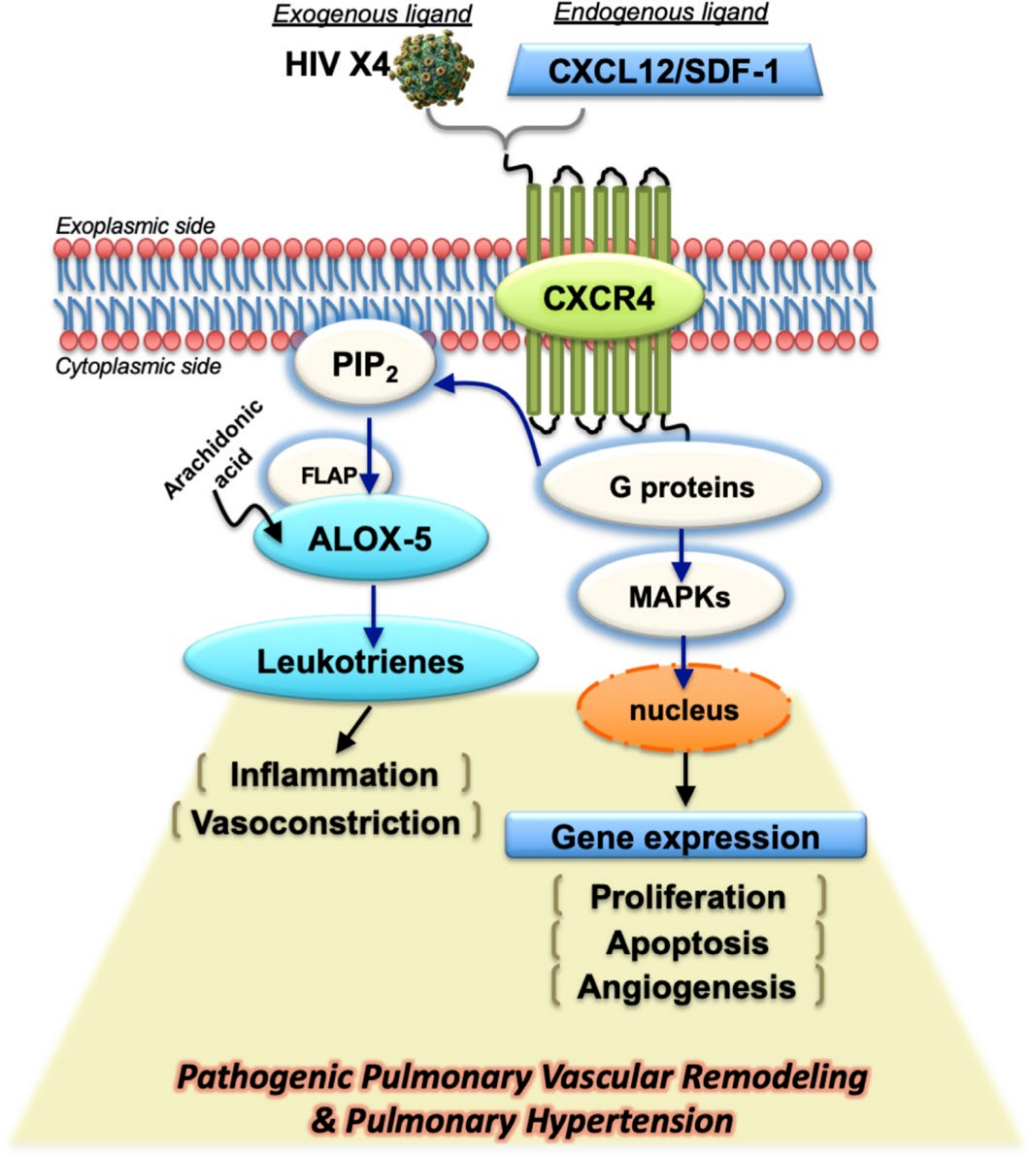
Hypothetical model for HIV-X4 viruses as pathogenic agents in Pulmonary Hypertension. Interaction of exogenous HIV with pulmonary endothelial cell CXCR4 receptors triggers CXCR4 cell signaling cascades involving activation of G proteins, mitogen-activated protein kinases (MAPK), followed by changes in gene expression leading to increased proliferation and aberrant apoptosis. Phosphatidylinositol 4,5-bisphosphate (PIP_2_) is an upstream intermediate between ALOX5 and CXCR4. Recruitment of 5-lipoxygenase-activating protein (FLAP) and arachidonic acid leads to increased production of 5-lipoxygenase, coded by ALOX5 gene. The concomitant activation of PIP_2_ may favor the formation of inflammatory vasoconstrictive molecules such as 5-lipoxygenase and leukotrienes in the absence of viral infection. The over-representation of X4 variants together with increased density of CXCR4 receptors on the cell membrane may create microenvironmental conditions most likely favoring the noxious outcomes of X4 viruses in the pulmonary vasculature.

Intriguingly, phosphatidylinositol 4,5-bisphosphate (PIP_2_) is an upstream intermediate in the ALOX5 and CXCR4 signaling pathways^79^. Hence, based on our findings and the scientific literature, we propose a mechanistic model to suggest that HIV *gp120* variants X4 and R5 have a differential impact on the pulmonary vasculature (Fig. 6). Highlighted by the increased presence of X4 viruses in PAH patients, interactions of X4 viruses with endothelial CXCR4 co-receptors are likely driving pulmonary vascular inflammation. Subsequently, this chain of events leads to increased production of ALOX5 which catalyzes the metabolism of arachidonic acid to produce potent chemotactic agents LTB_4_ and cysteinyl LT group (LTC_4_, LTD_4_, and LTE_4_), all of which increase vascular permeability and vasoconstriction and are broadly associated with PAH.

Our proposed model, based on the finding of ALOX5 as a downstream mediator relevant to HIV-PAH, directs towards new therapeutic opportunities in the field. Efforts to cure HIV have included the development of viral fusion inhibitors. On the one hand, maraviroc blocks the CCR5 receptor, which also improves PAH^24^. On the other hand, blocking CXCR4 has been useful in experimental PAH and promising in cancer^80, 81^ but its side effects still preclude its clinical use^82^. Therefore, HIV effects mediated by the CXCR4 pathway still remain largely unapproached by current antiretroviral drug strategies. Blockers of ALOX5 are currently being used in humans as a safe maintenance therapy for chronic asthma. Based on our findings, we foresee the supplementation of ALOX5 antagonists with current antiretroviral drug cocktails as new therapeutic venues to further investigate in order to protect those HIV-infected patients with circulating X4 variants from deadly respiratory complications like pulmonary arterial hypertension.

The finding of over-represented HIV X4 viruses in PAH invite to the discussion about the implications of viral isoforms, receptor binding and subsequent trigger of signaling pathways and pathogenic outcomes. The endothelium, as in other microenvironments, display HIV chemokine co-receptors unevenly. For instance, there is a relatively low expression of CCR5 receptor but a high density of chemokine receptor CXCR4 at the translational, protein and functional levels ^76, 77, 83–85^. Hence, our finding that X4 viruses are over-represented in patients with PAH implicate that microenvironmental conditions in the pulmonary vasculature most likely favor the noxious outcomes of X4 viruses, because of the higher predominance of CXCR4 receptors as their interactive partners.

In essence, our study has uncovered X4 viruses as potential agents in the pathophysiology of HIV-PAH. To the best of our knowledge, this is the first report linking HIV *gp120* genotype to a pulmonary disease phenotype. Noteworthy, this study does not discount the pathogenic roles of gp120^R5^. Previous studies have demonstrated that gp120^R5^-induced oxidative stress results in HIF-1α dependent up-regulation of PDGF-BB, which may lead to HIV-PAH^86^. While HIV gp120 is an insulting viral protein to the pulmonary vascular endothelium, R5 is different than X4, with differential impact on pulmonary vascular cells.

## Materials and methods

### Human subjects

We analyzed peripheral blood samples from 39 HIV-infected individuals enrolled in the NIH/NHLBI Lung HIV Studies at the University of California—San Francisco (UCSF) and University of Colorado (CU-Anschutz) who underwent evaluation for PAH. Diagnoses of HIV-associated PAH were made based on the standard diagnostic algorithm, which includes Doppler echocardiography and right heart catheterization (RHC) if echocardiography suggested PAH. PAH diagnoses were defined as mean pulmonary artery pressures (mPAP) > 25 mm Hg and pulmonary capillary wedge pressures (PCWP) ≤ 15 mm Hg via RHC. Informed consenting individuals with documented HIV infection longer than six months and able to provide anecdotal or medical evidence of HIV medication history were included in this study. Patients with PAH-associated co-morbidities such as left-heart disease, severe respiratory diseases, chronic thromboembolic pulmonary hypertension, connective tissue disease, congenital heart disease, or portal hypertension were excluded. These studies were approved by the Colorado Multiple Institutional Review Board, the Committee on Human Research at UCSF, and the Emory University Institutional Review Board following the guidelines and regulations as implemented by the NIH/NHLBI Lung HIV Data Safety and Monitoring Board.

### Animal subjects

Male Fischer 344 wild-type and HIV Transgenic (Tg) rats were bred in the animal facility at the Atlanta VA under a 12:12 light–dark cycle and fed *ab libitum*. All studies were completed in compliance with protocols approved by the Atlanta VA Animal Care and Use Committee and following the NIH guidelines for animal care and housing. The HIV Tg rats for this study were generated from established lines of an HIV provirus. This HIV Tg rat model was developed at UMD using the NL4-3 *gag/pol* HIV transgene^87^. The HIV Tg rat line has proviral DNA with deleted *gag* and *pol* but intact *env* and *tat, nef, rev, vif, vpr,* and *vpu* accessory genes^88, 89^. HIV transgene expression has been detected in the intestines, and at low levels in kidney, lymph nodes, lung and spleen^87, 90^. We used hemizygous, 7–9 month-old rats in this study.

### HIV *gp120* sequence analyses

The HIV-*gp120* C2-V4 region was amplified from peripheral blood mononuclear cells (PBMC) in duplicate or triplicate by polymerase chain reaction (PCR) using nested primers^91, 92^. The final products (~ 525 bp for *gp120*) were pooled and cloned into pCR2.1 (Life Technologies); approximately 20 clones were sequenced per patient. Nucleotide sequences of *gp120* C2-V4 region were aligned against HIV reference sequences in Los Alamos National Laboratory (HIV databases, https://www.hiv.lanl.gov) and edited using Geneious v.5.4.6 for Mac (Geneious Pro Biomatters Ltd.^93^. The amino acid sequence of the V3 region of the HIV envelope protein was used to predict the HIV co-receptor usage using Geno2Pheno (co-receptor) 2.5 web-based application^94^ (Max Planck Informatics Institute). All the co-receptor usage predictions were based on a 10% false positive rate (FPR), unless otherwise indicated; FPR values ≤ 10% predicted HIV-X4 strains, while FPR values > 10% predictive of HIV-R5 viruses.

### Cell culture

Human pulmonary artery endothelial Cells (HPAEC) were purchased from Lonza Walkersville and cultured in EBM-2 basal medium and supplements, respectively, as provided by the manufacturer, at 37 °C in a humidified incubator at 5% CO_2_ atmosphere; for experiments performed at UC-Anschutz, the CO_2_ injection was adjusted to Denver’s elevated altitude. Assays were performed on cells in passage 5 or 6, at 70–80% confluence. For cultures with HIV glycoproteins, we used the HIV recombinant proteins R5 (gp120^BaL^) and X4 (gp120^IIIB^) at 500 ng/mL concentration, obtained through the NIH AIDS Reagent Program, Division of AIDS, NIAID, NIH: HIV gp120_BaL_ recombinant protein from DAIDS, NIAID; and HIV gp120_IIIb_ recombinant protein from ImmunoDX, LLC. Monocyte-derived macrophages (MDM; purchased from Dr. Howard Gendelman) were cultured at 37 °C with 5% CO_2_ in DMEM containing 10% human serum, L-glutamine, penicillin–streptomycin and macrophage colony stimulating factor (M-CSF) for 7 days. MDM (5 × 10^6^) were infected with HIV_ADA_ (clade B) at a multiplicity of infection (MOI) of 0.1 for 1 h. Following infection, MDM were resuspended in medium devoid of M-CSF and cultured for 14 days with media changes every 3 days^95^. HIV p24 levels were measured in media by ELISA (Advanced BioSciences Laboratories). Lung bronchial epithelial cells (HBE4-E6/E7-C1) were purchased from ATCC and cultured in basal media and supplements in the Keratinocyte Serum-Free Kit (Gibco) as recommended by the manufacturer. Phospho ERK/AKT and JNK levels were measured under serum-containing conditions.

### Gene expression analyses

Cell-associated total RNA was extracted from HPAEC using a miRNeasy Mini Kit (Qiagen) or RNA Bee (AMSBIO). The quality of the extracted RNA was assessed in a R6K ScreenTape (Agilent Technologies). RNA (1.5 microg) was either combined with random nanomer primers (Ambion), dNTPs (New England Bio-Labs) and nuclease-free water for reverse transcription for further amplification using gene-specific primers or reverse transcribed using the RT^2^ First Strand Kit (Qiagen) and analyzed using the Endothelial Cell Biology PCR array (Qiagen). All transcripts were detected using SYBR Green I (Molecular Probes, Inc). Transcripts were normalized to the housekeeping gene, β-actin, β-2-microglobulin, and ribosomal protein large P0. Expression changes were determined using the 2^-ΔΔCt^ method.

### Analysis of HIV co-receptor expression

We measured the expression of HIV chemokine co-receptors CCR5 and CXCR4 by flow cytometry in HPAEC co-cultured in the presence of uninfected or HIV-infected T lymphocytes. The cell line SupT1 (NIH AIDS Reagents Program) was infected by spinoculation with pNL43-based HIV-X4 or HIV-R5 constructs (a kind gift from Dr. Yasuko Tsunetsugu-Yokota)^96, 97^ and added to HPAEC cultures for 48 h. Cells were stained with fluorescent antibodies againt cell surface markers with the following antibodies: anti-human CXCR4 (Pharmingen # 555976), and anti-human CCR5 (Pharmingen # 555993) as per standard immunostaining procedures. Cells were acquired in a BD Accuri C6 Flow Cytometer System and analyzed using FlowJo v.10.1r1 analysis software.

### Protein analyses

HPAEC lysates were subjected to Western blot analyses using primary antibodies for ALOX5 and GAPDH that were purchased from Cayman Chemical Company and Sigma-Aldrich, respectively. Proteins were visualized using chemiluminescent anti-goat or anti-rabbit secondary antibodies using a LI-COR imaging system. Bands for protein of interest were quantified by densitometry and normalized to GAPDH levels within the same lane. Leukotriene-specific ELISA kits (Cayman Chemical) were used to measure leukotriene levels in HPAEC and in animal bronchoalveolar lavage fluid (BALF). Phospho ERK/AKT and JNK levels were analyzed after treating bronchial epithelial HBE4 cells with 500 ng/mL of recombinant HIV glycoproteins in 6-well plates for 5, 10, and 60 min. The ERK signaling pathway as tested in whole cell lysates by using antibodies against pERK, tERK, pAKT, tAKT, pSAPK-JNK, tSAPK-JNK and beta actin. HIV p24 ELISA (Advanced BioScience Laboratories) was used to measure p24 levels in media from HIV-infected MDM. All ELISAs were performed according to manufacturer’s instructions.

### Cell proliferation and apoptosis assays

Cell proliferation was assessed by nuclear counts using a red fluorescent dye; apoptosis was analyzed by measuring the activity of caspases 3/7 using a green fluorescent substrate. Specifically, proliferating cells were seeded on a 96-well plate at ~ 30% confluence and incubated for 24 h to let the cells adhere. On the next day, cells were treated with the HIV glycoproteins. We also added a caspase 3/7 substrate reagent, IncuCyte Kinetic Caspase-3/7 Apoptosis Assay Reagent to measure apoptosis, or NucLight Rapid Red Reagent to count nuclei, as per the manufacturer protocol, in a final volume of 200 uL per well. Cells were incubated at 37 °C, 5% CO2 and imaged in an IncuCyte ZOOM system. Reagents and imager were manufactured by Essen Biosciences.

### Statistical analysis

Student’s t-test analyses were used for 2-group comparisons. One-way ANOVA with Tukey’s posttest was used for the comparison of multiple groups. All experiments using cell cultures were repeated at least twice on different primary cell batches, and samples were run in duplicate or triplicate. Statistical significance was defined as *p* < 0.05 and all graphs are expressed as mean ± SEM. For patient clinical correlations, we stratified the dataset by pulmonary hypertensive phenotype and used logistic regression to model the effects of predicted tropism and CD4 count above or below 200 cells/ul on PAH as outcome. To account for correlation among observations taken from the same subject over time, subject-specific random intercepts were included in the model. All statistical analyses were performed using GraphPad Prism or SAS 9.3 software.

## Data Availability

The datasets generated during and/or analysed during the current study are available from the corresponding author on reasonable request.
